# Geometric relationships of neural and bony landmarks of the cranial base

**DOI:** 10.1007/s12565-025-00886-7

**Published:** 2025-07-30

**Authors:** Jonathan A. Millard, Kaivon Kouhestani, Amanda Swaak, Alexandra Luna, Elina M. Baltins, Chelsea J. Bengson, Benjamin Mann, Sally S. Greenberg, Ryan Baukhages

**Affiliations:** 1 Edward Via College of Osteopathic Medicine—Virginia Campus, 2265 Kraft Drive SW, Blacksburg, VA 24060 USA; 2 Department of Surgery, Virginia Commonwealth University School of Medicine, Richmond, VA USA; 3 Department of Surgery, Carilion Clinic—Virginia Tech School of Medicine, Roanoke, VA USA; 4 Department of Psychiatry, University of Maryland Medical Center, Baltimore, MD USA

**Keywords:** Cranial base, Craniometrics, Geometric morphometrics, Morphology, Skull base

## Abstract

The skull base serves as structural support for the brain and as a conduit for neurovasculature. Traditional morphometric methods have intrinsic limitations which make wholistic assessment of this anatomy challenging. Here we applied geometric morphometric techniques to address the problems associated with traditional morphometric strategies for evaluating skeletal and soft tissue components of the skull base. A Microscribe® i+ 3-D digitizer was used to register the location of 20 3-D skeletal and neural landmarks on the skull base of 80 anatomic body donors (mean age 83.0 ± 10.1 years). Linear measurements of neurocranial dimensions were collected with traditional methods. A principal component analysis (PCA) was applied to Procrustes aligned coordinates of the 3-D data. The first principal component accounted for 34.24% of the variation and was associated with changes in clival width and sella turcica length. Shape changes associated with PC1 scores correlated with configuration size (centroid size) (*R*^2^ = .532, *p* < .001). PC2 (12.85%) was associated with isolated changes in clival width driven by the jugular tubercles. Results related to PC3 (11.10%) showed variation in skull base flattening. PC2 and PC3 results were correlated with neurocranial length and measurements of cranial width. Skull base size emerged as a principal driver of skull base shape. The jugular tubercle and surrounding foramina represented another locale of variation. Finally, our results support the idea that a wide range of non-pathologic skull base flattening may exist, as this shape axis was a major contributor to the variation seen in the sample.

**Supplementary Information:**

The online version contains supplementary material available at https://doi.org/10.1007/s12565-025-00886-7.

## Introduction

The skull base is a bony and cartilaginous feature that serves as a support and interface between the brain and extracranial elements. Bones of the skull base contain both proper foramina and openings formed at the junctions between participating bones. These openings communicate neurovascular content that are carefully considered during surgical approaches to the cranial base. 

There have been numerous morphometric strategies to describe attributes of the skull base and related cranial nerve relationships. Often these efforts aimed to assess local anatomic features. For example, clival morphometry has been evaluated in different geographic populations, age categories, and clinical groups (Dufton et al. 2011; Chaurasia et al. 2017; Belgin et al. 2021; Serindere et al. 2021). Iskra et al. reported a meta-analysis of 91 studies describing the morphometry of the sella turcica (Iskra et al. 2023). Individual foramina have also been the subject of analysis. Qualitative and quantitative features of the jugular foramen have been reported using both dry skulls and clinical imaging (Hatiboğlu and Anil 1992; Prades et al. 1994; Idowu 2004; Gupta et al. 2014). Significant structural variability in the size and shape of the internal acoustic meatus have also been demonstrated (Thomsen et al. 1981; Sakashita and Sando 1995; Tomura et al. 1995; Farahani et al. 2007; Marques et al. 2012). In addition to skeletal components, the relationships of cranial nerves with basicranial features have also been explored. Variables in these studies have ranged from looking at the number of intradural cranial nerve roots to the topographic relationship of these roots with other anatomic locations (Wysiadecki et al. 2015; Kuruc et al. 2021). 

Often, these isolated variables are evaluated collectively in an effort to inform surgical planning; however, there are intrinsic limitations to these methods (Aljuboori et al. 2020). Typical morphometric measurement techniques like linear dimensions, angles, and surface areas inevitably sacrifice some amount of biologic information, generally due to morphological complexity. In recent decades, geometric morphometric (GM) techniques have been developed to circumvent many traditional biometric problems by assessing variation in global shape using generalized Procrustes superimposition applied to homologous landmarks or semilandmark schemes (Goodall 1991). Often, this approach offers greater capture of the morphology resulting in a more comprehensive assessment of the variation in the shape. Although GM methods are usually seen in systematics and related fields, recent interest has included more clinical applications (Rutland et al. 2021; Katsube et al. 2022; Perera et al. 2024). Our primary aim is to apply landmark-based GM methods to evaluate geometric relationships of skeletal and soft tissue features of the skull base and relate the resulting shape variables to more traditional craniometric measurements. We believe this will enhance our understanding of the patterns of skeletal and neural variability seen in the sphenopetroclival region.

## Materials and methods

### Sample

The brains from 80 formalin-fixed anatomic body donors (44 female, 36 male, mean age 83.0 ± 10.1 years) were removed to reveal the cranial fossae. Donors with extensive damage to skeletal or neural elements related to the landmarking protocol were excluded from the sample. The protocol was reviewed and approved by the Edward Via College of Osteopathic Medicine Institutional Review Board (IRB Record #: 2022-014). The authors hereby confirm that every effort was made to comply with all local and international ethical guidelines and laws concerning the use of human cadaveric donors in anatomic research.

### Landmarking protocol and measurements

**Fig. 1:**
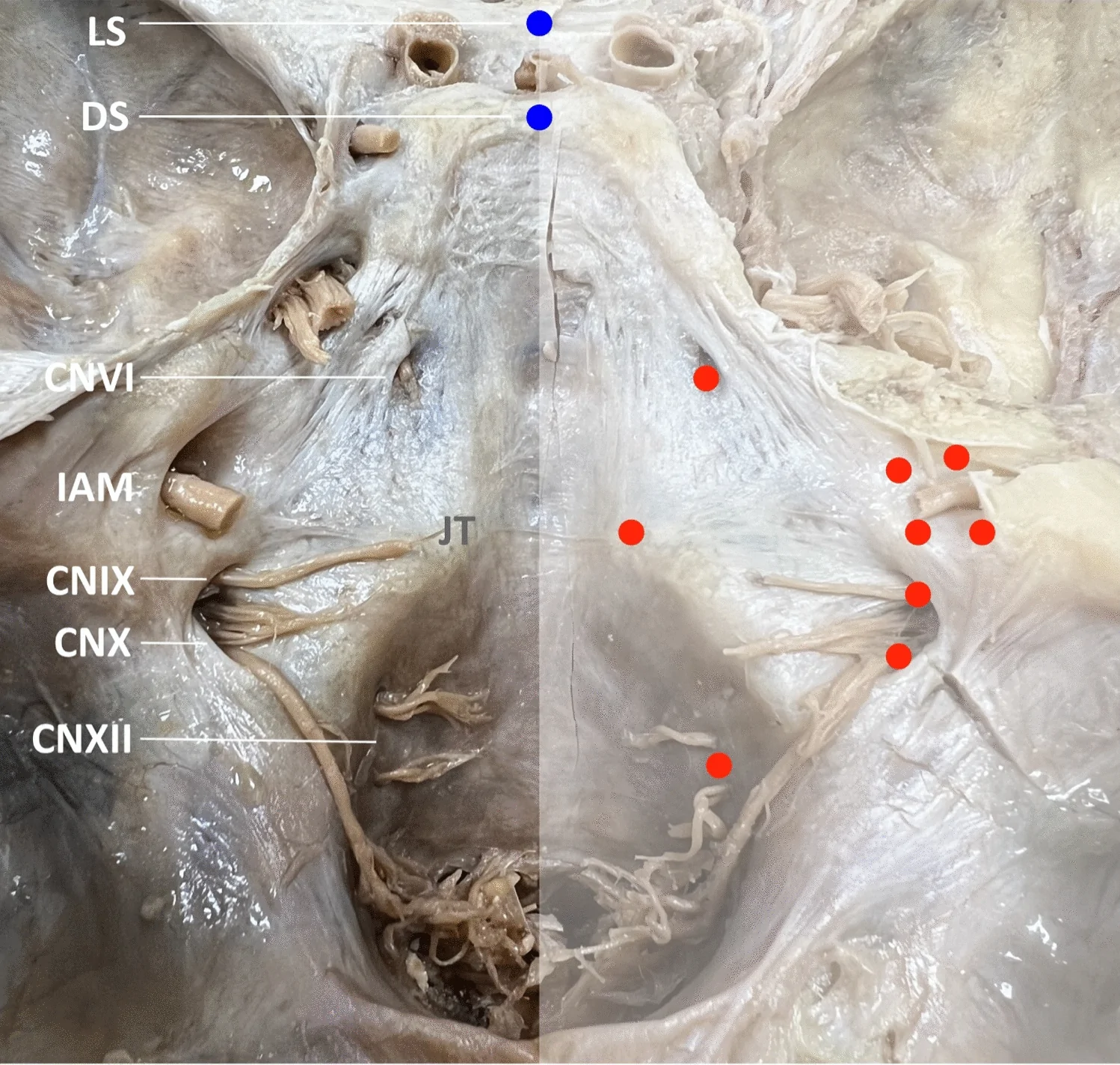
Middle and posterior cranial fossae with landmark locations. Blue points indicate unpaired midline landmarks; red points indicate paired landmarks. LS—limbus sphenoidale, DS—dorsum sella, IAM—internal acoustic meatus, JT—jugular tubercle

A Microscribe® i+ 3-D portable coordinate measuring machine was used to register the location of 20 3-D skeletal and neural landmarks on the skull base of the middle and posterior cranial fossae. The landmarking protocol consisted of two unpaired midline landmarks and nine paired landmarks (Table 1, Fig. 1). In cases where a landmark was not collected due to damage during the dissection, these individuals were excluded entirely from the analysis. The primary factors influencing landmark selection were reliability in location, adequate spread to ensure reasonable coverage of the area of interest, and clinical salience. Although not strictly bony landmarks, the dural meati of cranial nerves represent extremely high-fidelity landmark locations as very little interpretation is needed to determine their precise 3-D location. These in contrast with landmarks like the jugular tubercle which may exhibit more inter-landmarker variability due to differences in landmarker interpretation. Collecting these extremal points are useful when clearly defined and understood by landmarkers as they allow for the capture and ultimate description of more complex surfaces and shapes (Wärmländer et al. 2019).

**Table 1:** Landmark descriptions and definitions

Landmark	Abbreviation	Definition
Limbus sphenoidale^a^	LS	Midsagittal point along the limbus of the planum sphenoidale
Dorsum sellae^a^	DS	Midsagittal point on the superior aspect of the dorsum sellae
Abducens nerve	CN VI	Point where the abducens nerve penetrates the dura mater
Glossopharyngeal nerve	CN IX	Point where the glossopharyngeal nerve penetrates the dura mater; estimated as the highest point of the dural opening in cases of a shared dural meatus with vagus nerve
Vagus nerve	CN X	Most inferior point of vagus nerve dural penetration
Superior IAM	SIAM	Superior-most boundary of the opening of the internal acoustic meatus
Lateral IAM	LIAM	Lateral-most boundary of the opening of the internal acoustic meatus
Inferior IAM	IIAM	Inferior-most boundary of the opening of the internal acoustic meatus
Medial IAM	MIAM	Medial-most boundary of the opening of the internal acoustic meatus
Jugular tubercle	JT	Superomedial-most point on the jugular tubercle
Hypoglossal nerve	CN XII	Point where the hypoglossal nerve penetrates the dura mater; midway point between dural meati in the case of multiple dural openings

*IAM* internal acoustic meatus

^a^Indicates unpaired landmark

**Fig. 2:**
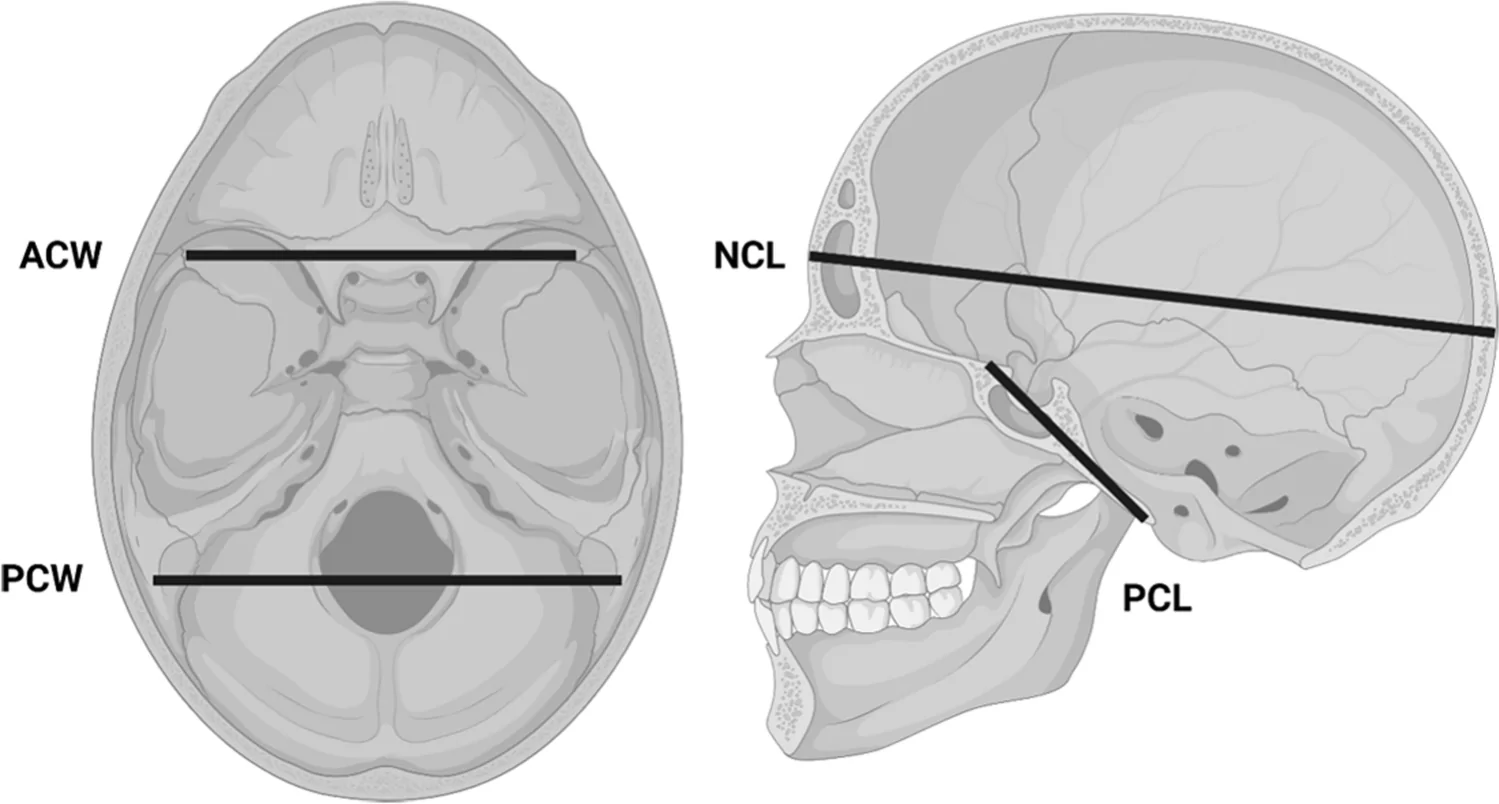
Cranial measurements, ACW—anterior endocranial width, PCW—posterior endocranial width, NCL—Distance from glabella to opisthocranion, PCL—posterior cranial length. *Created with BioRender.com*

In addition to collecting 3-D data, many other measurements were taken, including, neurocranial length, anterior endocranial width, posterior endocranial width, and posterior cranial length (Table 2, Fig. 2). These metrics mirror more traditional craniometric measurements and were designed to explore the relationships between global linear dimensions and 3-D shape variables. Neurocranial length measurements were collected with cranial spreading calipers and the remaining cranial measurements were taken with Neiko 01407A electronic calipers. As a way of estimating general body size, the length of the right ulna was taken as a proxy for height (Madden et al. 2012; Forman et al. 2014; Sarma et al. 2022).

**Table 2:** Linear measurements descriptions and definitions

Measurement	Abbreviation	Definition
Neurocranial length	NCL	Distance from glabella to opisthocranion
Anterior endocranial width	ACW	Endocranial distance between the left and right epipteric confluence at the frontal–sphenoid–temporal junction
Posterior endocranial width	PCW	Endocranial distance between the left and right petro-squamo-mastoid junction of the temporal bone
Posterior cranial length	PCL	Distance from the midline limbus sphenoidale to basion
Right ulna length	Ulna	Distance from superior tip of the right olecranon process to the tip of the ulnar styloid process

### Analysis

The raw landmark coordinates were exported into MorphoJ (v1.07a). The cranial base has mirrored bilaterally symmetry, so the symmetric component of shape was chosen to assess global differences in the skull base. Effects of fluctuating or directional asymmetry are associated with local environmental stress and biomechanical adaptations, which may vary between populations (Deleon 2007; Bigoni et al. 2013). Therefore, emphasis was placed on the symmetric variation seen in the skull base. A generalized Procrustes superimposition was performed to translate coordinates to a common centroid, scale to a common centroid size for each individual, and rotate each configuration such that the sum of the squared distances between landmarks and their mean locations is minimized. A covariance matrix was developed for the Procrustes aligned coordinates and a principal component analysis (PCA) was performed to explore variation in the landmark configuration. Lollipop graphs were created to gain a superficial understanding of the major eigenvectors contributing to shape variation. In addition to principal component (PC) scores, centroid size was collected for each individual. Centroid size is the most common scale metric assessed in GM methodology and is frequently used to evaluate the relationship of shape variables with size (Rohlf and Slice 1990).

Differences between males and females were assessed using independent samples t-tests for the manually collected measurements of the cranium and ulna (*α* = 0.05). Homoscedasticity between groups were assessed with Levine’s test; any test violating this assumption was converted to a non-parametric test. Regression analyses were performed to evaluate the relationship between PC scores and the manual measurements. In addition, regression analyses were used to assess the relationship between the major axes of shape variation and centroid size.

### Visualization techniques

The first four PC scores were plotted to assess variation. After preliminary examination of the lollipop graphs, the first three were pursued for their potential biologic significance. The Procrustes aligned coordinates of the symmetric component of shape were imported into RStudio (2024.12.1) and a PC analysis was ran using the geomorph package. The mean landmark configuration along with the maximum and minimum scores for the first three PCs were plotted using the rgl package.

## Results

### Geometric morphometric outcomes

**Fig. 3:**
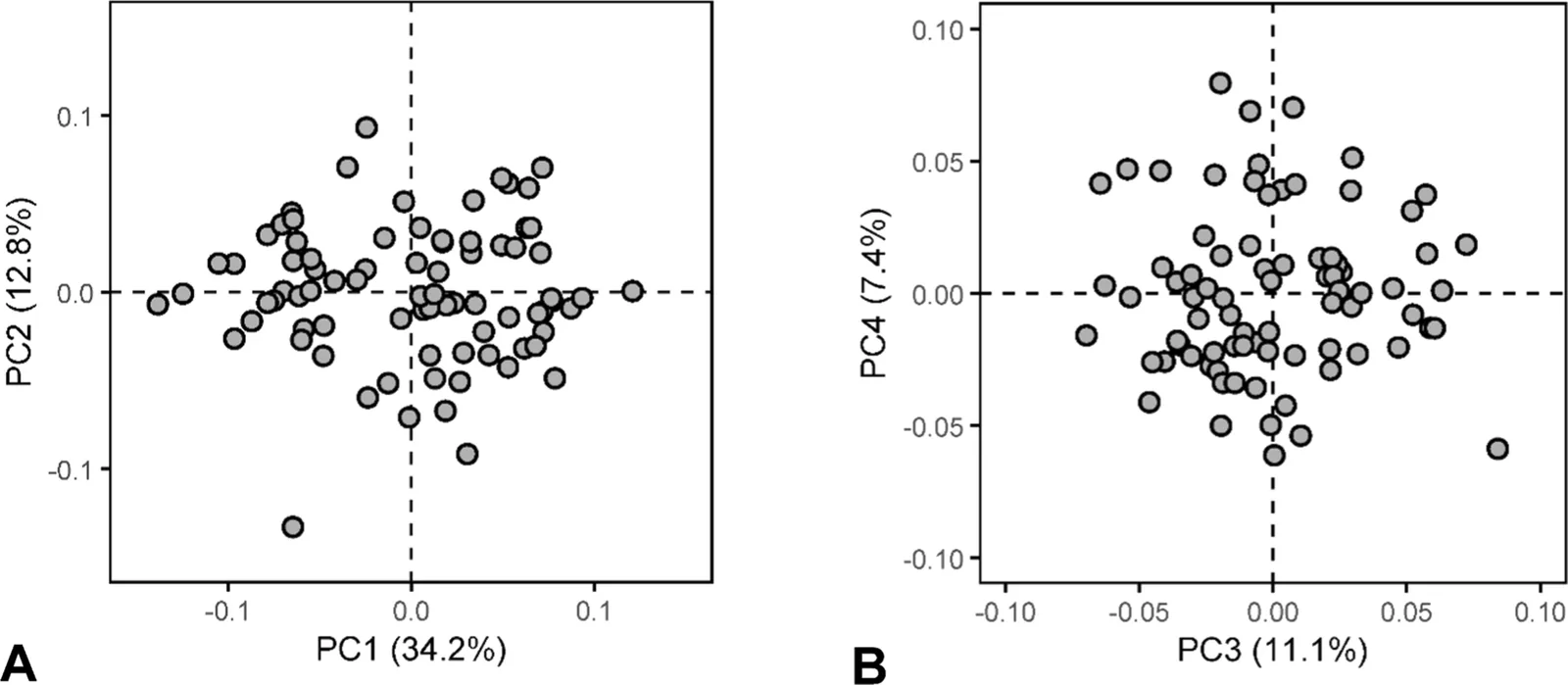
PC scores for the first four principal components. **A** PC1 vs PC2 scores; **B** PC3 vs PC4 scores. The individual selected for CT scanning is represented by the red point in the scatterplots

**Fig. 4:**
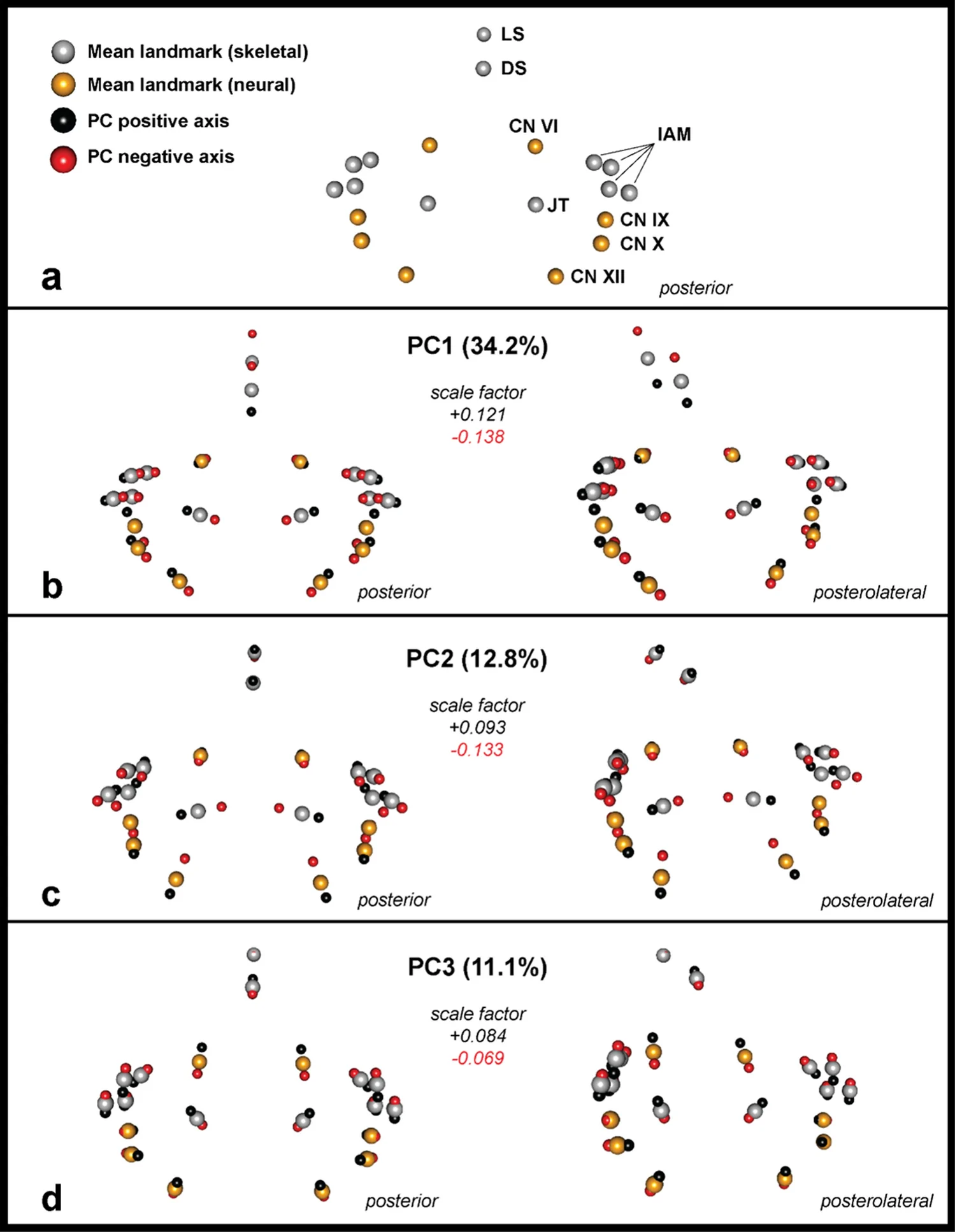
Results of PCA. **a** Mean landmark configuration; **b** PC1; **c** PC2; **d** PC3. LS—limbus sphenoidale, DS—dorsum sellae, IAM—internal acoustic meatus, JT—jugular tubercle

The PCA revealed 27 PCs with the first ten accounting for 86.5% of the variation in the sample and the first three PCs accounting for 58.2% of variation in the sample (Table 3, Fig. 3). The first PC (34.24%) exhibited changes in the height of parasellar features of the sphenoid bone with concomitant changes in clival width. More positive PC1 scores were associated with a shortened sella turcica and a relatively wider clivus at the level of the jugular tubercles, with mid-clival widening outpacing the lateral displacement of the dural meati for CN VI and CN XII (Fig. 4B, Supplement 1). These more positive scores along PC1 resulted in a less vertical and more laterally angled inferior petrosal sinus; in addition, lateral displacement of the jugular tubercles also resulted in the approximation of the internal acoustic meatus with the pars nervosa of the jugular foramen.

**Table 3:** First ten principal components with relative contributions to variation in the sample

PC	Contribution (%)	Cumulative contribution (%)
1	34.244	34.244
2	12.846	47.090
3	11.100	58.189
4	7.365	65.554
5	5.634	71.188
6	4.004	75.192
7	3.619	78.812
8	2.989	81.801
9	2.517	84.318
10	2.208	86.526

*PC* principal component

In contrast to PC1 results, the shape changes associated with PC2 (12.85%) include clival widening without notable changes in the height of the parasellar features (Fig. 4C, Supplement 2). More negative scores along PC2 were associated with clival narrowing being associated with a more elongated petrous apex, as evidenced by anteromedial displacement of the internal acoustic meatus and dural porus for CN VI. Configuration changes associated with PC3 (11.10%) were associated with flattening of the features (Fig. 4D, Supplement 3). Negative scores along PC3 were associated with a more coronally oriented landmark arrangement and positive scores were associated with a flatter cranial base, elongated in the anterior–posterior plane. Eigenvalues beyond PC3 were considerably lower and lacked meaningful distinction, therefore the first three components were selected for further analysis.

### Results of manual measurements

The results of the cranial measurements were similar to the findings of other reports, with the average neurocranial length being 168.5 mm (SD = 11.0) (Catapan et al. 2015; Ku et al. 2020). This value has been shown to have some variability based on population and demographics (Lee and Park 2008). Our measures of anterior and posterior endocranial width are less common, but were necessary deviations from more conventional measures of cranial width as all the calvaria were cut in advance of the study. Specifically, calvaria were cut (as part of a separate education curriculum) circumferentially in a typical fashion to access the brain and cranial fossae, often extending below the level of the squamosal suture. These previous cuts made accurately establishing euryon by assessing maximum cranial breadth inconsistent or impossible in many cases. Clival length from the dorsum sellae to basion is not an uncommon measurement; here we elected to extend this measurement from basion to the limbus on the body of the sphenoid bone as this encompasses the components of the sphenoid bone captured in the 3-D data collection. Unsurprisingly, these measurements were significantly larger in males within the sample (Table 4).

**Table 4:** Measurements collected of the cranium and ulna

Variable	Mean (*SD*) Female (mm)	Mean (*SD*) Male (mm)	*df*	*t*-stat	Sig (two-tail)
NCL	166.0 (10.5)	171.6 (11.0)	78	−2.328	**0.023**
ACW^a^	118.65 (6.21)	123.31 (8.31)	61	−2.759	**0.008**
PCW	129.34 (11.05)	134.57 (9.82)	78	−2.218	**0.029**
PCL	69.93 (5.15)	75.06 (4.99)	77	−4.468	**< 0.001**
Ulna	259.3 (20.8)	296.6 (21.8)	77	−7.764	**< 0.001**

**Bold** items are significant

*NCL* neurocranial length, *ACW* anterior endocranial width, *PCW* posterior endocranial width, *PCL* posterior cranial length, *Ulna* right ulna length

^a^Indicates calculation where equal variances are not assumed based on Levine’s test for equality of variances

### Relationship between shape variables and manual measurements

**Fig. 5:**
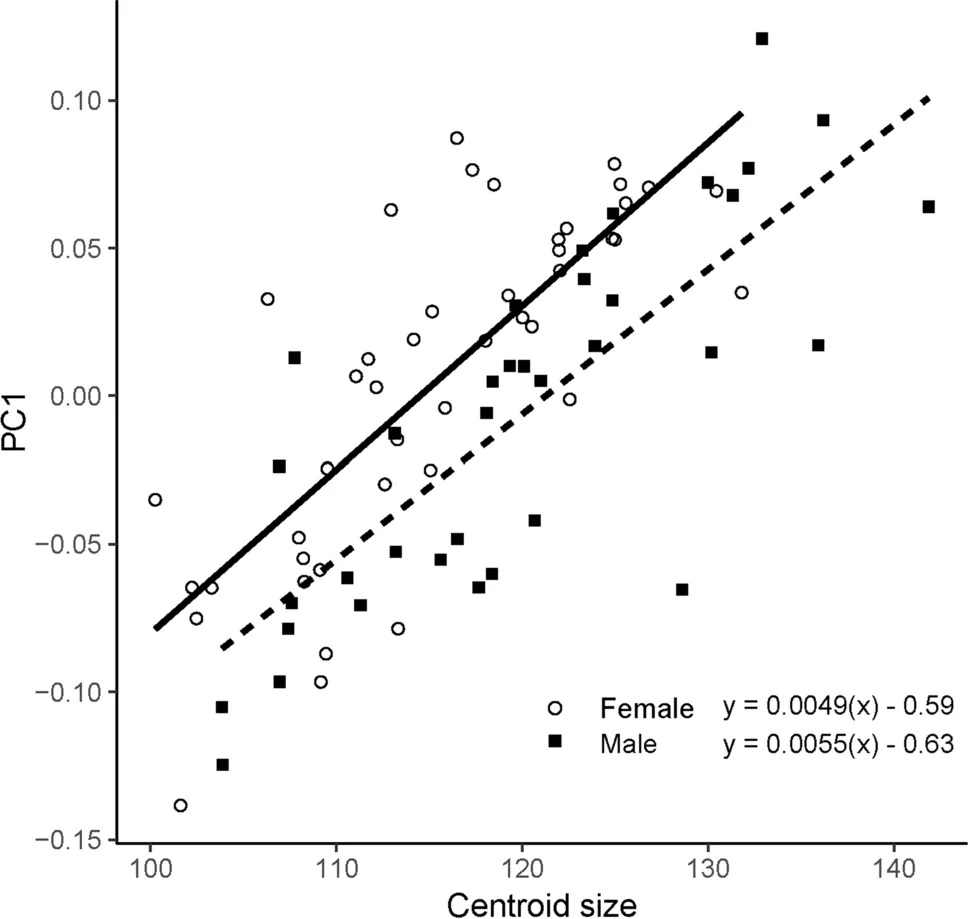
Relationship between PC1 scores and centroid size

The results of the regression analyses for PC1 scores revealed significant relationships between PC1 and centroid size (*p* < 0.001), as well as PC1 and posterior cranial length (*p* < 0.001) (Table 5). Overall, 53% of the variance along PC1 was explained by the size of the configuration (*R*^2^ = 0.532) (Fig. 5). The average male centroid size (*n* = 36, $\overline{x}$ = 119.9, *SD* = 9.9) was slightly larger than female centroid size (*n* = 44, $\overline{x}$ = 115.5, *SD* = 8.0) (*t* = 2.22, *p* = 0.029). Scores for PC2 and PC3 had significant relationships with neurocranial length (*p* = 0.007 and *p* < 0.001, respectively) and posterior endocranial width (*p* = 0.008 and *p* = 0.002, respectively). There was also a significant relationship between PC3 scores and anterior endocranial width (*p* = 0.015). There were no significant relationships between scores along the PC2 and PC3 axes and centroid size. These results suggest that size is the primary driver of shape variation in the sample. Although not statistically significant, PC1 scores had a marginal correlation with ulnar length which was not seen for PC2 or PC3 scores. There were no meaningful relationships between shape and age.

**Table 5:** Regression analyses examining the relationship between principal component scores and manual measurements

Variable	PC1 scores (34.24%)			PC2 scores (12.85%)			PC3 scores (11.10%)		
	*R*^2^	*F*	*p*	*R*^2^	*F*	*P*	*R*^2^	*F*	*p*
NCL	0.031	2.523	0.116	0.088	7.547	0.0**07**	0.161	14.950	< 0.0**01**
ACW	0.013	1.010	0.318	0.003	0.244	0.622	0.074	6.164	0.0**15**
PCW	0.010	0.791	0.376	0.088	7.501	0.0**08**	0.115	10.171	0.0**02**
PCL	0.188	17.833	< 0.0**01**	0.034	2.724	0.103	0.018	1.445	0.233
Ulna	0.040	3.167	0.079	0.005	0.393	0.533	0.002	0.193	0.662
CS	0.532	88.709	< 0.0**01**	0.003	0.212	0.647	0.003	0.266	0.607
Age	0.002	0.148	0.701	0.016	1.305	0.257	0.007	0.585	0.447

**Bold** items are significant

*NCL* neurocranial length, *ACW* anterior endocranial width, *PCW* posterior endocranial width, *PCL* posterior cranial length, *CS* centroid size

## Discussion

### Interpreting shape findings

The primary finding of our study is that skull base size is a major driver of skull base shape, as evidenced by the relationship between PC1 scores and centroid size. The interpretation of skull base size as a principal driver of shape is further reinforced by the relationship between our measurement of posterior cranial length and PC1 scores (*p* < 0.001). Larger configurations tend to have a relatively wider clivus with a shortened sella turcica. Smaller configurations are associated with a narrower clivus and elongated skull base along PC1, resulting in a sella turcica that is stretched along the midline. Our findings suggest the relationship between shape and size along PC1 seems isolated to the skull base, independent of meaningful relationships with cranial width or cranial length. Similar craniometric studies in the field of anthropology use analogous methods to assess skull features, particularly as it pertains to comparative relationships or variations in anatomically modern human populations (Singleton 2002; Dedouit et al. 2017). Often these relationships center on neurocranial globularity and relationships with the facial skeleton (Lieberman et al. 2002). Our results along PC1 combine many of these previously described techniques by assessing the relationship between 3-D geometric skull base configurations and more global measurements of the cranium.

The findings along PC2 (12.85%) shared many characteristics with findings along PC3 (11.10%), with neither exhibiting the extreme height relationships between the clivus and parasellar features seen along PC1. Instead, changes along PC2 showed similar variability in clival width independent of a meaningful effect on the sella turcica. Clival changes along PC1 were a uniform widening/narrowing of almost all posterior fossa features, while clival widening associated with PC2 seemed to be driven more by the jugular tubercles. Negative scores along PC2 were associated with more horizontally oriented jugular tubercles and a narrower overall clivus, while more positive scores were associated with more vertically positioned jugular tubercles and a wider overall clivus. On PC2, more horizontally oriented jugular tubercles and a narrower clivus were associated with an elongated petrous temporal bone, whereas more vertically oriented jugular tubercles and a wider clivus were associated with a shorter petrous temporal bone. More vertically oriented jugular tubercles/wider clivus were also associated with a much more inferior hypoglossal canal position. Suslu et al. found extreme variability in the morphology of the jugular tubercles, noting seven different classifications (Suslu et al. 2014). Here we are able to relate variation in the jugular tubercle with consequences associated with local bony arrangement. These findings are particularly relevant to surgical approaches to the posterior cranial fossa, as the jugular tubercle is often a target of resection to enhance exposure and acquire adequate surgical corridors (Mintelis et al. 2006; Fernandez-Miranda et al. 2012). Our findings along PC2 suggest that more vertically oriented jugular tubercles are more closely approximated to neural elements of the jugular foramen and farther from the hypoglossal canal. This may influence preoperative planning and enhance clinical outcomes.

The third PC of interest—PC3 (11.10%)—has similar temporal bone features as PC2, with variability in petrous elongation. While there were slight changes to clival width along PC3, the angles of the jugular tubercles were relatively constant. Our primary finding along PC3 is variation in the angle of skull base features. Negative scores on PC3 were associated with a less flattened and more vertically oriented skull base, while positive scores were associated with generalized flattening of the skull base. This result is consistent with platybasic descriptions of the skull base, which is an increase in basal angle of greater than 143° (Koenigsberg et al. 2005; Ferreira and Botelho 2019). Larger basal angles are associated with congenital conditions like Chiari malformation and other craniofacial anomalies (Cogan and Barrows 1954; Nachmani et al. 2022). Acquired forms of platybasia have been described in Paget’s disease and osteogenesis imperfecta (Poppel et al. 1953; Frank et al. 1982). A meta-analysis by Ferreira and Botelho found that normal basal angles can range between 104° and 129° (Ferreira and Botelho 2019). Our findings along PC3 support the idea that variation in basal angle represents a meaningful contribution to overall skull base variation, independent of congenital or acquired pathologic conditions.

### Limitations

There are many limitations to our study that should be considered. The sample was relatively small, with the majority of the individuals being of recent European ancestry. These are not unusual issues with cadaveric research in the United States but should be acknowledged. Considerations for age-related skeletal changes make these results less generalizable to a younger population. The landmarking scheme was robust but not invulnerable to landmarker bias and some degree of systematic error. In addition, assuring a robust landmarking protocol using reliable homologous landmarks, inevitably—in the case of physical/non-virtual landmark collection—will result in interpretation of intervening anatomy which is not technically landmarked. These may be common and reasonable inferences but must be exercised judiciously as to not make overreaching conclusions about the data.

## Conclusions

In summary, our primary finding is that skull base size is a principal driver of skull base shape, which varies primarily by narrowing/widening of the clivus with related lengthening/shortening of the sella turcica. Interestingly, this seemed largely independent of cranial length or cranial width, suggesting a local allometric relationship in the cranial base. The second primary finding is that skull base widening may occur independently of major morphologic changes to the sella turcica, driven primarily by mediolateral fanning of the jugular tubercles. This should be considered in neurosurgical contexts, as the jugular tubercles are frequently targeted to access the posterior cranial fossa. The final primary finding of the variation in the degree of skull base flattening as the third largest contributor to overall shape variation in skull base shape supports the idea that a wide range of non-pathologic configurations may exist in the population. 

## Supplementary Information

Below is the link to the electronic supplementary material.

Supplementary file1 (MP4 2980 KB)

Supplementary file2 (MP4 3167 KB)

Supplementary file3 (MP4 2530 KB)

## Data Availability

The raw data used to generate this report may be accessed at the online repository figshare - (https://doi.org/10.6084/m9.figshare.27886287.v1).
